# Intraoperative Fibrinous Reaction During Phacoemulsification With Posterior Chamber Intraocular Lens Implantation

**DOI:** 10.7759/cureus.46968

**Published:** 2023-10-13

**Authors:** Jawaher J Alwatban, Ghadah M AlQahtani, Turki Alotaibi, Syed Raheman

**Affiliations:** 1 Fellowship and Residency Training Program, King Khaled Eye Specialist Hospital, Riyadh, SAU; 2 College of Medicine, King Saud Bin Abdulaziz University for Health Sciences, Riyadh, SAU; 3 Opthalmology, King Khaled Eye Specialist Hospital, Riyadh, SAU

**Keywords:** fibrin reaction, intraoperative, cataract surgery, phacoemulsification, anterior chamber, fibrinous reaction

## Abstract

This is a case report of a 67-year-old female who underwent phacoemulsification and posterior chamber intraocular lens (IOL) implantation and developed a rare fibrinous reaction intraoperatively. During surgery, the patient experienced poor dilation and iris tissue prolapse. Phacoemulsification and IOL insertion into the capsular bag were performed. A fibrinous reaction was noticed at the end of surgery and was managed excellently with triamcinolone. Postoperatively, the patient achieved visual acuity of 20/20, with no flare or fibrinous reaction observed on slit lamp examination. This case report highlights the possible related mechanisms of such an event, as well as clinical management and response to treatment. To reduce the risk of complications, close follow-up and prompt initiation of anti-inflammatory therapy are essential. Further studies are needed to investigate the predisposing factors for developing fibrinous reactions during cataract extraction.

## Introduction

The fibrinous reaction, also known as the pupillary membrane reaction, is thought to be an inflammatory response caused by a breakdown of the blood-aqueous barrier (BAB). Fibrinogen is among its most prominent components, which triggers the synthesis of intraocular fibrin [1]. Several factors may contribute to the development of this complication, surgical traumas, intraocular inflammation (e.g., uveitis, scleritis), vascular disorders (e.g., Coats, Eales), systemic disorders (e.g., diabetes) and intraocular tumors (e.g., retinoblastoma, uveal melanoma) [2,3].

The fibrinous reaction occurring in the intraoperative period in an adult patient during cataract surgery is a rare event, while the postoperative fibrinous reaction is relatively common, with reported incidence rates ranging from 5% to 12% [4]. Intraoperative anterior chamber fibrinous reactions have been reported with Descemet membrane endothelial keratoplasty [1,5]. It is essential to distinguish between fibrinous reaction and toxic anterior segment syndrome. The fibrinous reaction is characterized by a lack of pain, exuberant swelling, redness, significant corneal edema, anterior chamber reaction, or anterior vitreous reaction [6]. Topical steroids have proven highly effective in resolving fibrin bands within a few weeks without causing permanent damage. This is when anti-inflammatory therapy is initiated promptly, and close monitoring is maintained. We report a case of a 67-year-old female patient with a known case of hypertension who was admitted to our institution for phacoemulsification and intraocular lens (IOL) insertion. She experienced a rare complication of a fibrinous reaction occurring intra-operatively.

## Case presentation

A 67-year-old Middle Eastern female presented with decreased vision in both eyes, with the right eye experiencing a pronounced decline over the past few years. She had no history of eye pain, redness, or systemic symptoms. The patient had well-regulated hypertension without any concomitant systemic conditions or autoimmune disorders. Her medical history was otherwise unremarkable, with no pertinent personal or familial medical history. On examination, her best corrected visual acuity was 20/400 in the right eye and 20/60 in the left eye, with both eyes having normal intraocular pressure. Slit-lamp examination of the right eye revealed a shallow anterior chamber with no cells or flare observed. A white cataractous lens was visible, and no view of the fundus was possible. No retinal detachments or other pathologies were noted on B-scan ultrasonography. As for the left eye, the anterior segment was quiet with nuclear sclerosis and cortical cataracts. The posterior pole was normal with no signs of ocular illness. The patient's random blood glucose was 6.7 mmol/L, and inflammatory markers such as lymphocytes and monocytes were borderline elevated, with signs of microcytic anemia. All other inflammatory markers and laboratory results were within the expected normal range. During the pre-operative evaluation, the patient showed fair dilation in both eyes.

**Figure 1:**
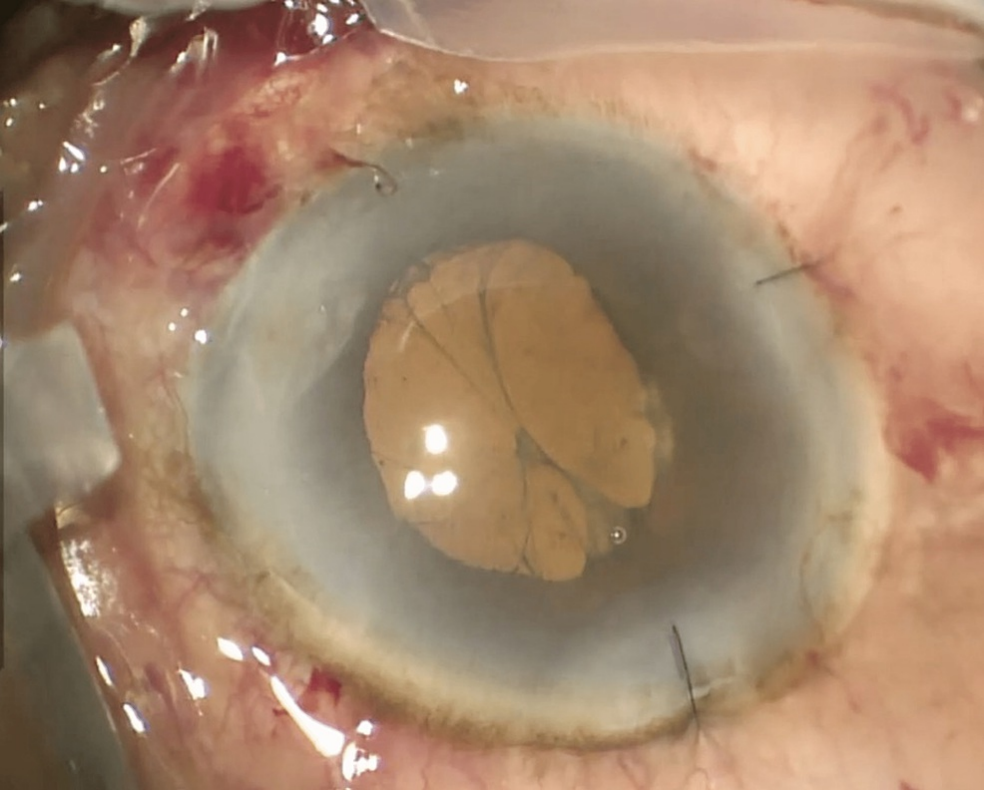
Intraoperative Fibrinous Reaction. Thick, rope-like fibrin cords in the anterior chamber intraoperatively during phacoemulsification with posterior capsular intraocular lens (IOL) insertion. The cords resolved within two hours post-operatively after immediate intracameral injection of triamcinolone.

**Figure 2:**
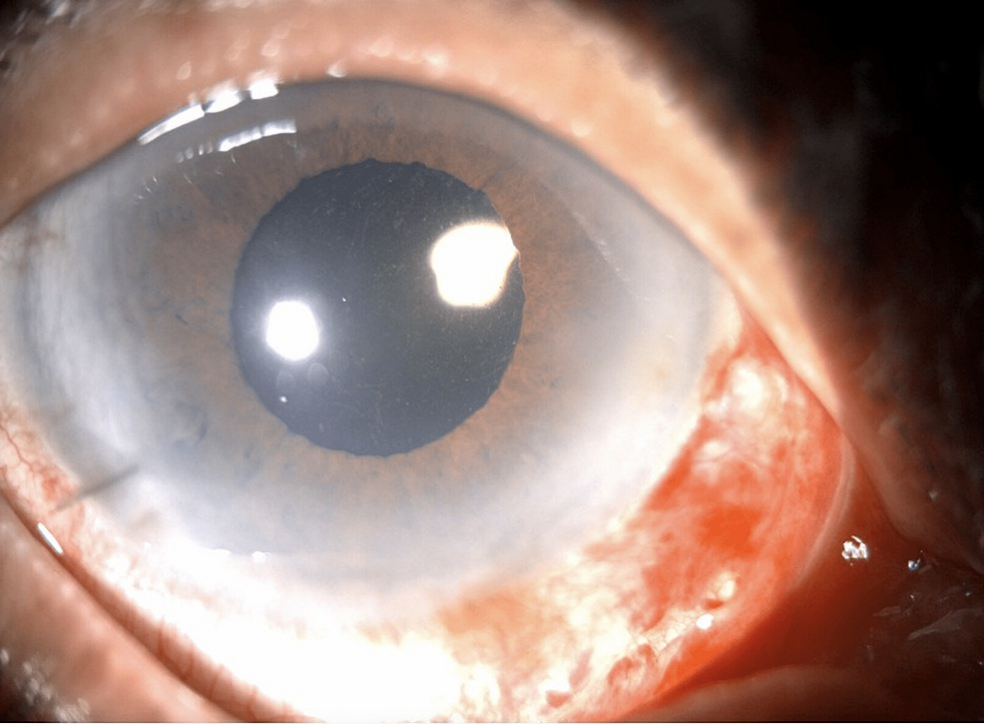
A slit lamp photo of the operated eye in the first post-operative visit. A complete resolution of the fibrinous reaction was noted in the first post-operative visit.

The patient underwent phacoemulsification and IOL insertion in her right eye. During surgery, the iris tissue prolapsed through the main wound with attempts to reinsert the iris back in its position the iris was manipulated which led to pupil constriction. Iris hooks were inserted to aid in pupil dilation. Phacoemulsification was performed, and the remaining cortical material was removed by irrigation and aspiration. Viscoelastic material (OVD) was used including DisCoVisc (Alcon, Fort Worth, TX, USA) to coat the endothelium and ProVisc (Alcon) to inflate the capsular bag. A single-piece Acrysof IQ IOL (Model SN60WF; Alcon) was inserted into the capsular bag. At the end of the procedure, a fibrinous reaction was noted (Figure 1). Intracameral cefuroxime 1.0mg/0.1mL, triamcinolone 1.0mg/0.1mL, and sub-conjunctival decadron 2mg/0.5m were injected. The patient showed no fibrinous reaction when seen two to three hours post-operatively. The operative eye's visual acuity and intraocular pressure were 20/20 and 18-mm Hg by pneumotonometer on day one after surgery, with no cells or flare observed on slit lamp examination (Figure 2). The patient was started on prednisolone acetate tapering over the course of six weeks, moxifloxacin, and cyclopentolate. After a six-week follow-up, the patient was seen in the clinic with no cells or flare observed. The anterior chamber was deep and quiet. Fundus examination was normal with no signs of ocular inflammation. The patient was happy with her vision.

## Discussion

Intraoperative fibrinous reaction in the anterior chamber is an infrequent complication that ophthalmologists may encounter during cataract surgery. To our knowledge, this is the first reported case of an intraoperative fibrinous reaction occurring in the intraoperative period in an adult patient during cataract surgery. Alternatively, reports have indicated instances of intraoperative anterior chamber fibrinous reaction occurring during Descemet membrane endothelial keratoplasty [1,5].

One of the postulated mechanisms that could explain this incidence of fibrinous reaction in our case is the disruption of the BAB. Possible related factors accountable for the blood-aqueous breakdown include surgical trauma, resulting in a variable inward movement of inflammatory cells and blood plasma constituents such as proteins, cytokines, and growth factors [2]. In relation to our presented case, surgical trauma is perhaps the leading factor in the intraoperative formation of fibrous reactions. The mere manipulation of iris tissue has been associated with increased inflammatory response [7]. Trinh et al. present a series of five cases where an unexpected fibrin reaction occurred in the anterior chamber during Descemet membrane endothelial keratoplasty (DMEK). Among these cases, one patient experienced iris prolapse during cataract surgery while undergoing combined DMEK-cataract surgery. Based on their findings, the researchers strongly advise surgeons to exercise caution to maintain the integrity of the iris during surgery. They emphasize that any form of iris damage, including iris prolapse, can heighten the risk of breakdown in the BAB and contribute to increased intraoperative inflammation [5]. Another factor predisposing to surgical trauma is improper use of viscoelastic material or certain intraocular irrigation solutions can cause mechanical trauma to the iris and lead to the release of inflammatory markers [2]. The inflammatory response is caused by prostaglandin release, and cell necrosis of the iris and/or ciliary body leading to increased vascular permeability and the influx of white blood cells and protein. Prostaglandins play a major role in disrupting the blood-aqueous barrier and can be activated by various stimuli, including paracentesis [8-10]. IOLs are made of inert material and do not usually cause inflammation by themselves if placed inside the capsular bag. However, it has been implied that IOL implantation may play a role in minimal and short-lasting BAB alteration [11].

Postoperatively, intracameral injection of 25 µg 25mcg/0.1mL recombinant tissue plasminogen activator (t-PA) is an effective treatment modality for fibrinous reactions following cataract surgery [12,13]. In our case, immediate intracameral injection of triamcinolone demonstrated excellent results [14]. The self-limiting nature of the fibrin reaction and its accelerated response to steroid treatment are characteristics of an immune complex reaction [15].

## Conclusions

In conclusion, this case highlights the need for careful preoperative assessment and intraoperative monitoring to prevent, diagnose, and treat such complications promptly. It is also essential to start anti-inflammatory therapy promptly to avoid further damage to the eye. Close follow-up of the patient is necessary to monitor the response to treatment. Further studies are needed to elucidate this condition's pathophysiology and identify the possible predisposing factors for developing fibrinous reactions during cataract surgery.
